# Attention-enhanced segmentation network for automated cerebral microbleed detection and burden assessment

**DOI:** 10.3389/fnins.2026.1743039

**Published:** 2026-03-04

**Authors:** Kwon Hwi Cho, Jonghyun Jeon, Seonggyu Kim, Young Seo Kim, Yu-Mi Kim, Mi Kyung Kim, Min-Ho Shin, Insung Chung, Sang Baek Koh, Hyeon Chang Kim, Chae Jung Park, Jong-Min Lee

**Affiliations:** 1 Department of Artificial Intelligence, Hanyang University, Seoul, Republic of Korea; 2 Department of Neurology, Hanyang University College of Medicine, Seoul, Republic of Korea; 3 Department of Electronic Engineering, Hanyang University, Seoul, Republic of Korea; 4 Department of Preventive Medicine, Hanyang University College of Medicine, Seoul, Republic of Korea; 5 Department of Preventive Medicine, Chonnam National University Medical School, Gwangju, Republic of Korea; 6 Department of Occupational and Environmental Medicine, Keimyung University School of Medicine, Daegu, Republic of Korea; 7 Department of Preventive Medicine and Institute of Occupational Medicine, Yonsei Wonju College of Medicine, Wonju, Republic of Korea; 8 Department of Preventive Medicine, Yonsei University College of Medicine, Seoul, Republic of Korea; 9 Department of Radiology, Research Institute of Radiological Science, Yongin Severance Hospital, Yonsei University Health System, Yongin, Republic of Korea; 10 Department of Biomedical Engineering, Hanyang University, Seoul, Republic of Korea

**Keywords:** ARIA-H, attention mechanism, CBAM, cerebral microbleeds, segmentation

## Abstract

**Introduction:**

Cerebral microbleeds (CMBs) are small hemorrhagic lesions visible as hypointense foci on susceptibility-sensitive MRI and are established biomarkers of stroke risk and amyloid-related imaging abnormalities (ARIA-H) in patients receiving anti-amyloid therapy. However, automated detection remains challenging because true CMBs closely resemble veins, calcifications, and susceptibility artifacts. This visual ambiguity results in a persistent precision–recall trade-off, where models optimized for high sensitivity tend to generate excessive false positives, while precision-focused models risk missing clinically relevant lesions. To address this limitation, we propose an attention-enhanced segmentation framework designed to suppress confounding activations while preserving lesion sensitivity.

**Methods:**

We developed RLK-UNet with Convolutional Block Attention Modules (CBAM), a single-stage encoder–decoder architecture that redefines skip connections as context-filtered pathways. The encoder incorporates large 13×13 residual local kernel (RLK) convolutions to capture broad contextual information for distinguishing spherical microbleeds from elongated vascular structures. CBAM modules are embedded in all skip connections to selectively enhance lesion-relevant features and suppress irrelevant background responses before feature fusion. The model was trained and evaluated on a multi-site dataset of 506 T2*-GRE and SWI scans, with lesion-level detection assessed using precision, recall, F1-score, and average false positives per scan. Subject-level burden estimation was further evaluated using ARIA-H severity intervals.

**Results:**

The proposed model achieved state-of-the-art lesion-level performance, with a precision of 0.891, recall of 0.887, F1-score of 0.887, and a markedly reduced false positive rate of 0.83 per subject. Five-fold cross-validation demonstrated stable performance with minimal variance across splits. In lesions ≤3 mm, the model maintained strong detection performance (F1-score 0.869) while effectively controlling false positives. Cross-modality evaluation between T2*-GRE and SWI confirmed robust generalization. Ablation studies verified that CBAM significantly improved precision while preserving sensitivity, and Grad-CAM visualizations demonstrated more spatially focused and clinically interpretable attention patterns. Subject-level CMB counts strongly correlated with ground truth (Spearman *ρ* = 0.93), and severity classification aligned with ARIA-H intervals.

**Conclusion:**

RLK-UNet with CBAM provides a robust and interpretable solution for automated CMB detection by directly addressing false-positive propagation through attention-guided skip connections. The framework achieves balanced precision and sensitivity within a single-stage architecture and demonstrates reliable subject-level burden estimation aligned with clinically meaningful ARIA-H categories. These findings support its potential application in vascular risk stratification and treatment monitoring in patients undergoing anti-amyloid therapy.

## 1 Introduction

Cerebral microbleeds (CMBs) are small (<10 mm) perivascular hemosiderin deposits that appear as punctate hypointensities on susceptibility-sensitive magnetic resonance imaging (MRI), most commonly associated with hypertensive arteriopathy and cerebral amyloid angiopathy (Greenberg et al., 2009; Cordonnier and van der Flier, 2011; Fazekas et al., 1999). Their presence has been linked to an increased risk of stroke, cognitive decline, and dementia, even in other asymptomatic individuals (Akoudad et al., 2016; Gregoire et al., 2009). Accurate detection of CMBs is therefore clinically critical, not only for assessing intracerebral hemorrhage risk in patients considered for anticoagulation therapy, but also for monitoring amyloid-related imaging abnormalities (ARIA-H), a frequent adverse event in anti-amyloid immunotherapy (Lovelock et al., 2010; Hampel et al., 2023). Consequently, the reliable and interpretable detection of CMBs is becoming increasingly important for vascular risk management and dementia care.

Early efforts to detect CMBs primarily relied on manual visual rating scales, such as the Microbleed Anatomical Rating Scale (MARS) and the Brain Observer MicroBleed Scale (BOMBS), in which radiologists assessed hypointense lesions on T2*-weighted GRE or SWI images (Gregoire et al., 2009; Cordonnier et al., 2009). While these methods were clinically trusted, they were limited by inter-rater variability, inefficiency, and low sensitivity to subtle or small lesions. The higher sensitivity of SWI further increased the number of visible CMBs, exacerbating the manual burden and motivating the development of automated approaches.

Traditional machine learning (ML) methods represented the first attempts at automation, leveraging handcrafted features related to intensity, shape, and anatomical location. For example, Barnes et al. and Kuijf et al. combined candidate detection with Random Forest classifiers, while Ghafaryasl et al. used a support vector machine trained on shape and contextual features (Barnes et al., 2011; Kuijf et al., 2013; Ghafaryasl et al., 2012). These approaches demonstrated the feasibility of automated detection but were hampered by poor generalizability across different imaging conditions and susceptibility to false positives in complex anatomical regions. To overcome these limitations, deep learning–based models were introduced, particularly 3D convolutional neural networks (CNNs) that could exploit richer spatial context. Dou et al. pioneered a 3D patch-based CNN that improved sensitivity and better distinguished CMBs from confounding structures such as vessels and calcifications (Dou et al., 2016).

More recently, detection-based deep learning frameworks such as YOLO and SSD have been applied to CMB analysis. Al-Masni et al. proposed a two-stage pipeline combining YOLOv2 with a CNN-based classifier (Al-Masni et al., 2020), while Myung et al. enhanced YOLOv2 with cerebrospinal fluid (CSF) filtering as a post-processing step to reduce false positives (Myung et al., 2021). Li et al. further developed an SSD-based model that leveraged ground truth annotations during training to enhance feature learning (Li et al., 2021). Although these detection-based approaches demonstrated efficiency, their reliance on bounding boxes limited their ability to capture the precise morphology of CMBs, which are typically small and spherical.

Segmentation-based approaches, particularly those built on U-Net and its variants, have therefore become the preferred choice for automated CMB detection. U-Net’s encoder–decoder architecture enables multi-scale feature integration and pixel-level localization, which is crucial for delineating small, low-contrast lesions. Fan et al. demonstrated the effectiveness of multi-slice U-Net inputs for improving detection accuracy (Fan et al., 2022), while Tsuchida et al. proposed SHIVA-CMB, a 3D U-Net trained on seven cohorts with diverse acquisition protocols, showing strong generalization across sites (Tsuchida et al., 2024). Wei et al. introduced MMOC-Net, a two-stage architecture combining U-Net and a Full-Resolution Network (FRN), highlighting the importance of multi-scale fusion and hybrid loss functions for capturing small-object sensitivity (Wei et al., 2022). Collectively, these studies confirm the value of segmentation-based methods, yet also reveal persistent trade-offs between sensitivity and precision. Models tuned for high recall often produce large numbers of false positives, whereas those optimized for precision risk missing clinically significant lesions.

A key limitation of conventional U-Net–based frameworks lies in their skip connections, which indiscriminately transmit both lesion-relevant and irrelevant high-frequency features from the encoder to the decoder. This results in vascular textures, calcifications, and noise being propagated alongside true CMB signals, inflating false positives and undermining precision. To address this challenge, we reconceptualize skip connections as selective, context-filtered pathways rather than raw conduits. Specifically, our proposed framework integrates residual local kernel (RLK) convolutions with large receptive fields to capture broad contextual cues, together with Convolutional Block Attention Modules (CBAM) embedded in all skip connections. The RLK encoder provides contextual information to disambiguate spherical CMBs from elongated vascular structures, while CBAM selectively filters features before fusion into the decoder (Son et al., 2023; Woo et al., 2018). This design suppresses the propagation of confounding responses and amplifies lesion-relevant signals, alleviating the precision–recall trade-off at its source.

Importantly, CBAM generates attention maps that provide transparent visual evidence of what the model emphasizes or suppresses, enhancing clinical interpretability and trust. Unlike prior two-stage or post-processing frameworks, our method achieves balanced improvements in sensitivity and precision within a single end-to-end architecture. Furthermore, we show that the model’s subject-level predictions align with ARIA-H severity intervals, underscoring its translational relevance for monitoring treatment in patients receiving anti-amyloid therapies.

In summary, this study makes three key contributions. First, we redefine skip connections as context-aware, attention-gated pathways that directly address the root cause of false-positive propagation in CMB detection. Second, we propose a single-stage architecture that integrates large-kernel RLK encoding with CBAM-based skip filtering, improving precision without compromising sensitivity. Finally, we demonstrate the clinical applicability of our framework through accurate subject-level burden estimation aligned with ARIA-H severity categories, highlighting its potential for real-world deployment in dementia care and treatment monitoring.

## 2 Methods

### 2.1 Data preprocessing

All input magnetic resonance imaging data underwent a standardized preprocessing pipeline to ensure consistency and improve model performance. First, N4ITK bias field correction was applied to reduce low-frequency intensity inhomogeneity, thereby enhancing tissue contrast and supporting more stable feature learning (Tustison et al., 2010). Subsequently, non-brain tissues such as the skull and surrounding fat were removed using the Brain Extraction Tool (BET) from FSL (Jenkinson et al., 2012). To optimize memory usage and computational efficiency, each image was tightly cropped around the brain region, and all slices were resampled to a fixed resolution of 512 × 512 pixels.

During training, various data augmentation techniques were applied to improve generalization and mitigate overfitting. Specifically, random geometric transformations including ±15° rotations, horizontal flipping, and vertical flipping were used. To further focus the training on relevant anatomical areas, slices were cropped around non-zero mask regions, ensuring that lesion-containing areas were included in the input. Additionally, bezier curve-based nonlinear intensity distortion was introduced to simulate contrast variability across subjects and scanners, thus improving the model’s robustness to imaging heterogeneity.

All images were standardized using z-score normalization to have zero mean and unit variance. The data were processed on a 2D slice-wise basis. For model input, each sample was constructed as a three-channel input by stacking the center slice with its immediate preceding and succeeding slices.

This input configuration is anatomically motivated, as cerebral microbleeds are small, focal lesions that typically extend across only a limited number of contiguous axial slices. Incorporating adjacent slices allows the model to capture local inter-slice continuity relevant to microbleed detection while remaining within a 2D-based framework. In contrast, using all slices along the z-dimension as input channels would introduce substantial variability in input dimensionality due to differences in slice thickness and acquisition protocols, potentially impairing generalization in multi-center datasets. The specific input configuration and how it integrates with the network are described in the following section.

### 2.2 Network architecture

**Figure 1:**
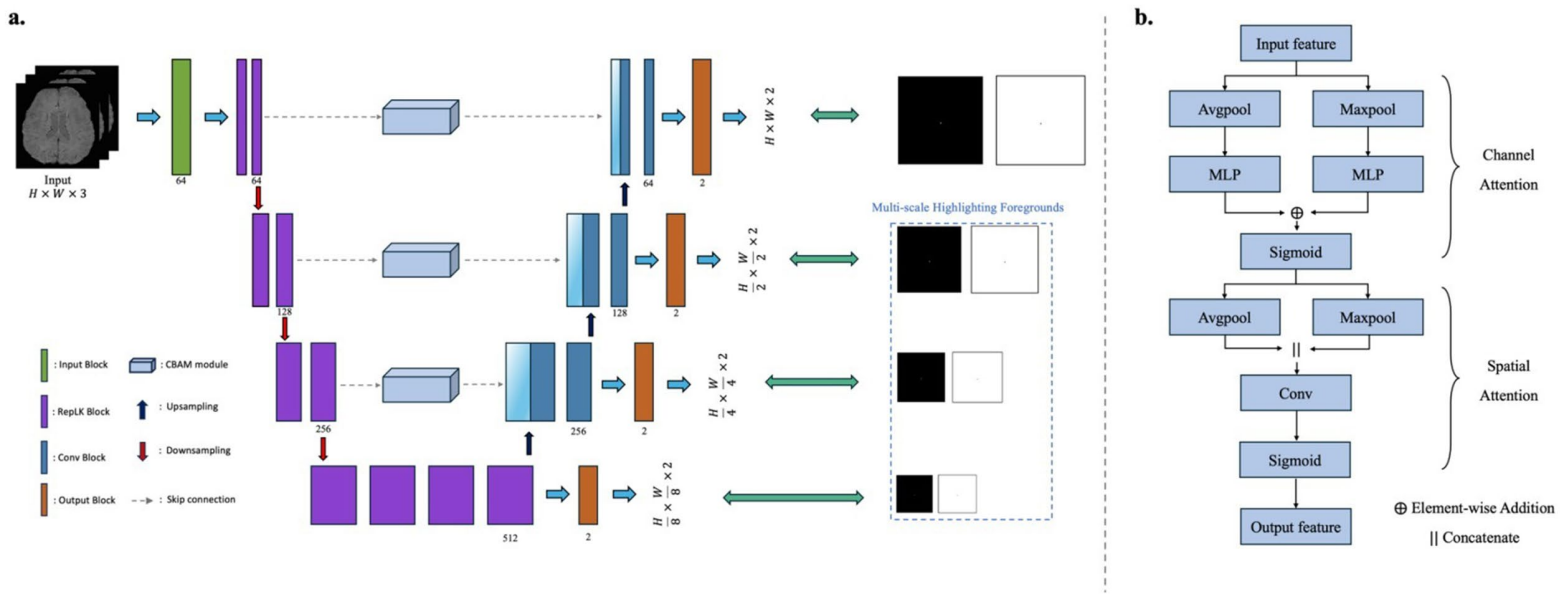
Overall architecture of the proposed RLK-UNet with CBAM for cerebral microbleed (CMB) segmentation. **(a)** The network adopts an encoder–decoder structure, where each encoder and decoder block incorporates residual local kernel (RLK) modules utilizing 13 × 13 convolutional filters to capture multi-scale context. CBAM, consisting of channel and spatial attention, is applied to all skip connections to enhance CMB-relevant features while suppressing irrelevant background activations. Attention gates are used to further refine the fused features passed from encoder to decoder. Multi-scale outputs are generated at three decoder stages to provide deep supervision and foreground highlighting. **(b)** The internal structure of CBAM is illustrated, showing the sequential application of channel attention (based on global average and max pooling followed by MLP and element-wise addition) and spatial attention (using convolution after concatenating pooled spatial maps). The model receives a three-channel input consisting of the target axial slice and its adjacent upper and lower slices, and outputs pixel-wise segmentation maps along with intermediate auxiliary outputs to improve learning effectiveness and interpretability.

As illustrated in Figure 1, the proposed model adopts a modified encoder–decoder architecture based on U-Net, designed specifically for the detection of cerebral microbleeds (CMBs). Although CMBs are three-dimensional anatomical entities, they typically appear as small, focal lesions spanning only a limited number of slices along the axial direction. Based on this observation, the network is built upon a 2D-based framework that effectively captures in-plane spatial features while maintaining robustness across heterogeneous imaging protocols.

The network integrates Residual Local Kernel (RLK) blocks and Convolutional Block Attention Modules (CBAM) to enhance discriminative feature learning and suppress irrelevant activations. The model is optimized for the segmentation of small and sparse CMBs, which are often challenging to distinguish due to their similarity to surrounding anatomical structures.

#### 2.2.1 Encoder: deep feature extraction with large receptive fields

The encoder consists of four stages, each composed of two or more convolutional blocks. Unlike conventional U-Net architectures that typically use 3 × 3 or 5 × 5 kernels, our RLK blocks utilize large 13 × 13 kernels. These wide convolutional filters allow the network to capture a broader receptive field, enabling the extraction of more extensive contextual information around potential lesions (Son et al., 2023). Given that typical cerebral microbleeds have a diameter of approximately 2–10 mm, corresponding to roughly 5–20 voxels at the 512 × 512 in-plane resolution used in this study, the 13 × 13 kernel was selected to encompass the average spatial extent of microbleeds and their surrounding context, facilitating discrimination from linear vascular structures. This is particularly beneficial for distinguishing true CMBs from confounding structures such as veins and calcifications. Each block employs group normalization and GELU activation, and downsampling between stages is performed using strided convolutions. To improve generalization, stochastic depth via droppath is applied with increasing drop rates across encoder levels.

#### 2.2.2 Decoder and attention-based skip connections

The decoder mirrors the encoder structure and consists of upsampling modules followed by convolutional refinement blocks. Each upsampling stage restores spatial resolution and fuses the corresponding encoder features via skip connections. To prevent the transfer of irrelevant features through the skip paths, we incorporate CBAM into every skip connection. CBAM refines the encoder features through two sequential modules: channel attention and spatial attention (Woo et al., 2018).

The channel attention map is computed as follows for an input feature map $F\in {\mathbb{R}}^{C\times H\times W}$ :


$$M_{c}(F)=\sigma (\mathrm{MLP}(\text{Avgpool}(F))+\mathrm{MLP}(\text{Maxpool}(F)))$$

where σ denotes the sigmoid function and MLP is a shared two-layer perceptron. The refined output $F^{\prime }$ is then passed through the spatial attention module, which computes:

$$M_{s}(F^{\prime })=\sigma (f^{7\times 7}([\text{Avgpool}(F^{\prime });\text{Maxpool}(F^{\prime })]))$$

The output from the spatial attention is multiplied with the input to generate the final attention-refined skip feature. In addition to CBAM, attention gates are applied in parallel to further enhance the spatial selectivity of the skip connections by conditioning on the decoder’s coarser outputs. This dual-attention mechanism helps suppress irrelevant activations and improves the segmentation precision.

#### 2.2.3 Multi-output deep supervision

The decoder generates auxiliary outputs at multiple stages to facilitate deep supervision. Each auxiliary output provides an intermediate lesion probability map at a different spatial scale. These outputs are used during training to propagate gradients more effectively, which is particularly important for cerebral microbleeds that are small in size and sparsely distributed.

By providing additional supervision signals at intermediate decoder levels, deep supervision helps stablize the training process and alleviates optimization difficulties commonly encountered in small-lesion segmentation tasks. While this strategy can lead to incremental improvements in final segmentation performance, its primary role in this work is to enhance training stability and improve gradient flow rather than to fundamentally alter the representational capacity of the network.

The final segmentation output is derived from the highest-resolution decoder stage and is supervised together with the auxiliary outputs using a combined loss function, with greater emphasis placed on the final output during training.

### 2.3 Loss function

To effectively supervise the segmentation of cerebral microbleeds (CMBs), which are typically small in size and sparsely distributed, we adopt a composite loss function that combines Dice Loss and Focal Loss. This formulation is designed to balance the need for high sensitivity with the suppression of false positives, addressing the class imbalance inherent in lesion segmentation tasks.

The Dice loss ${ℒ}_{\text{Dice}}$ directly optimizes the overlap between the predicted and ground truth masks, and is particularly effective for handling small lesion regions:


$$L_{\text{Dice}}=1-\frac{2\sum_{i}p_{i}g_{i}+\in }{\sum_{i}p_{i}+\sum_{i}g_{i}+\in }$$

where $p_{i}$ and $g_{i}$ denote the predicted probability and ground truth label for voxel i , respectively, and ϵ is a small constant for numerical stability.

To further enhance the model’s focus on hard-to-classify voxels, the Focal Loss ${ℒ}_{\text{Focal}}$ is employed, defined as:


$${ℒ}_{\text{Focal}}=-\alpha_{t}{\left(1-p_{t}\right)}^{\gamma }\log(p_{t})$$

where $p_{t}$ is the predicted probability corresponding to the true class, $\alpha_{t}$ is a balancing parameter, and γ is the focusing parameter. In our experiments, we set γ=2.0 to emphasize difficult samples and reduce the influence of well-classified background voxels.

Importantly, although subjects without cerebral microbleeds (zero-CMB cases) were not explicitly included during training, the use of Focal loss effectively mitigates the dominance of background voxels by down-weighting easy negative samples. This design encourages the network to focus on hard-to-classify lesion candidates ans duppress spurious false positives, thereby alleviating class imbalance even in the absence of explicit negative-only subjects during training.

The final training loss ${ℒ}_{\text{total}}$ is the weighted sum of Dice and Focal losses, computed across the main output and three auxiliary outputs from the decoder:


$$L_{\text{total}}=\sum_{k=1}^{4}(\lambda_{k}\cdot (L_{\text{Dice}}^{\left(k\right)}+L_{\text{Focal}}^{\left(k\right)}))$$

where $\lambda_{k}$ denotes the weight assigned to the output at the *k*-th decoder level. In our implementation, the weights were set to *λ* = {0.4,0.3,0.2,0.1} from the highest-resolution (final) output to the deepest auxiliary output, respectively. This weighting scheme places greater emphasis on the final prediction while allowing auxiliary outputs to contribute to training stability through deep supervision.

## 3 Experiments

### 3.1 Data

This study utilized a total of 506 brain MRI scans collected from three sources: the MICCAI2021 Challenge dataset, a multi-center cohort referred to as Cavas, and clinical data from Yongin Severance Hospital. The combined dataset includes both T2*-weighted Gradient Echo (T2*-GRE) and Susceptibility Weighted Imaging (SWI) sequences, reflecting real-world variability in acquisition protocols and scanner manufacturers.For evaluation, a test set comprising 72 subjects was constructed, while the remaining scans were used for training. Notably, the test set included subjects without cerebral microbleeds, ensuring a comprehensive assessment of the model’s ability to distinguish between positive and negative cases.

#### 3.1.1 MICCAI2021 challenge dataset

The MICCAI2021 dataset comprises 72 scans, of which 50 contain cerebral microbleeds, from three sub-cohorts: SABRE (11 scans), RSS (34 scans), and ALFA (27 scans) (Sudre et al., 2024). These images were acquired using either 1.5 T or 3 T MRI scanners (e.g., Philips Achieva, GE Discovery), using both 2D and 3D T2*-GRE protocols. Imaging parameters vary across cohorts, with slice thickness ranging from 0.8 mm to 3.0 mm and in-plane resolutions reaching up to 0.45 × 0.45 mm^2^. The ground truth CMB annotations were provided by the challenge organizers as part of the MICCAI2021 Small Vessel Disease Segmentation Challenge. Ethical approval for the use of this dataset can be referenced in the corresponding original study.

#### 3.1.2 Cavas dataset

CAVAS is a multi-cohort study focusing on normal aging in the rural population. Initial analysis was performed on a total of 270 scans, from which 154 scans exhibiting microbleeds were selected to form the CAVAS dataset, comprising five sub-cohorts: HY (21 scans), PH (55 scans), CN (43 scans), KB (22 scans), and GH (13 scans). These images were acquired using 3 T MRI scanners (e.g., Philips Achieva, Siemens Skyra) with a 2D T2*-GRE protocol. Imaging parameters include a slice thickness ranging from 4.0 mm to 7.0 mm and in-plane resolutions up to 0.80 × 0.80 mm^3^ to 0.89 × 0.89 mm^3^. The ground truth CMB annotations were provided by two neurologists participating in the in-depth aging survey research collaboration for rural-based cohorts. All data acquisitions were approved by the institutional review boards of the respective centers: Hanyang University (HYUIRB-202011-012), Chonnam National University (06–062), Keimyung University (2020–01-058), Wonju Yonsei University College of Medicine (CR320120), and Yonsei University Medical School (4–2020-0817).

#### 3.1.3 Yongin severance dataset

The Yongin Severance dataset consists of 374 brain MRI scans, of which 294 exhibit microbleeds. These scans were acquired using a 3D SWI protocol on two types of 3 T scanners: Philips Elition and Philips Ingenia CX. All scans were obtained using standardized acquisition parameters with a repetition time (TR) of 51 ms and echo time (TE) of 9.8 ms. The slice thickness was set to 2.0 mm. The in-plane acquisition resolution was 0.60 × 0.60 mm^2^, and the reconstructed resolution was refined to 0.45 × 0.45 mm^2^ with 1.0 mm slice thickness. The dataset was collected under the approval of the Yongin Severance Hospital Institutional Review Board (eIRB No. 9–2025-0165).Ground truth CMB annotations were established through a semi-automated procedure. An initial segmentation model trained on the MICCAI and CAVAS datasets was used to identify potential lesions. Experienced clinicians iteratively reviewed the outputs to eliminate false positives. For the final test set, manual segmentation was independently performed by board-certified neuroradiologists to ensure high annotation accuracy and reliability.

### 3.2 Evaluation metrics

To evaluate the lesion-level performance of the proposed segmentation model, four standard metrics were employed: precision, recall, F1-score, and the average number of false positives per scan ( $FP_{\mathrm{avg}}$ ). These metrics were selected to comprehensively assess both sensitivity and specificity, as well as the practical viability of the model in clinical applications.

All evaluation metrics were computed on a lesion-wise basis, where a detected lesion was considered a true positive if the Euclidean distance between its centroid and the nearest ground truth lesion was within 4 voxels. This threshold was selected to account for small lesion sizes and spatial localization uncertainty during annotation.

### 3.3 Settings

To implement and evaluate the proposed model, all experiments were conducted in a controlled computational environment summarized in Table 1.

**Table 1:** Summary of the training environment and hyperparameter configuration used in all experiments.

Software/Hardware	Model/Parameter
GPU	NVIDIA RTX A5000 Ada
Framework	Pytorch 1.13
Optimizer	AdamW
Initial learning rate	1e-4 (with warm-up from 1e-7)
Scheduler	Cosine Annealing Warmup Restarts
Epochs	1,000
Batch size	2

A warm-up strategy was applied at the beginning of training, where the learning rate was gradually increased from 1e-7 to 1e-4 over the first 10 epochs. After the warm-up phase, a cosine annealing schedule with warm restarts was used to adjust the learning rate dynamically throughout the remaining training epochs.

The model was trained using 2D slice-based input with a batch size of 2 for 1,000 epochs. All experiments were performed on a single NVIDIA RTX A5000 Ada GPU. At inference time, the proposed model required an average of 0.22 s per subject, measured on the test set. This runtime includes forward inference and post-processing for lesion detection.

Early stopping was not applied during training. Instead, model selection was performed based on validation performance within each fold of the K-fold cross-validation framework. Specifically, for each fold, the checkpoint achieving the highest validation F1-score was retained and used for final evaluation. Training and validation curves were monitored to ensure stable optimization, and the best-performing checkpoints were not consistently obtained at the final epoch, indicating no systematic overfitting during prolonged training.

## 4 Results and discussion

### 4.1 Quantitative evaluation

To assess the effectiveness of the proposed method, we conducted a comparative evaluation against existing cerebral microbleed (CMB) detection approaches, including both 3D CNN-based and segmentation-based models. The compared models were trained using the dataset employed in this study, with training conducted under conditions that reflected the environments originally proposed for each model. The evaluation metrics include precision, sensitivity (recall), F1-score, and the average number of false positives per subject ( $FP_{\mathrm{avg}}$ ). A detailed summary of these results is provided in Table 2.

**Table 2:** Comparison of detection performance between the proposed method and existing CMB detection approaches (2D lesion-level).

Method	Precision	Sensitivity (Recall)	F1-score	*FP_avg_*
Dou et al. (2016)	0.521	0.740	0.611	5.22
Fan et al. (2022)	0.714	0.765	0.739	2.35
Wei et al. (2022)	0.822	0.863	0.842	1.43
Tsuchida et al. (2024)	0.767	0.730	0.749	1.71
Ours	**0.891**	**0.887**	**0.887**	**0.83**

The bold values indicate the performance of the proposed model.

The proposed method demonstrates superior performance across all evaluation criteria. In terms of precision, our model achieves a value of 0.891, which is substantially higher than that of Dou et al. (0.521), Fan et al. (0.714), and even more recent methods such as Wei et al. (0.822) and Tsuchida et al. (0.767). This indicates that the proposed model is highly effective at reducing false positive predictions while maintaining accurate identification of true CMBs. Sensitivity also improves noticeably, reaching 0.887, which suggests the model is capable of detecting a large proportion of true lesions. Compared to other methods, which show recall values in the range of 0.730 to 0.863, this represents a meaningful gain in lesion retrieval ability.

The F1-score, which reflects the balance between precision and recall, is also the highest among all compared methods, with our model achieving a score of 0.887. This confirms that the model does not sacrifice sensitivity for precision or vice versa, but rather achieves a strong trade-off between both. Furthermore, the average number of false positives per subject ( $FP_{\mathrm{avg}}$ ) is significantly reduced to 0.83, while previous methods report considerably higher values, such as 5.22 for Dou et al., 2.35 for Fan et al., and 1.71 for Tsuchida et al. This dramatic reduction in $FP_{\mathrm{avg}}$ highlights the model’s robustness and its ability to suppress incorrect activations.

Overall, the consistent improvements in all metrics underscore the practical advantages of integrating CBAM into the RLK-UNet architecture. The attention mechanism contributes to better localization and discrimination of lesion-relevant features while reducing interference from non-lesion signals. These results strongly support the effectiveness of the proposed method for reliable and accurate detection of CMBs in complex neuroimaging data.

### 4.2 Cross-validation performance analysis

To evaluate the generalizability and robustness of the proposed RLK-UNet with CBAM, we conducted a 5-fold cross-validation on the dataset. Table 3 presents the detailed lesion-level detection performance for each fold in terms of precision, sensitivity (recall), F1-score, and the average number of false positives per subject ( $FP_{\mathrm{avg}}$ ). Across all folds, the model consistently demonstrated high detection accuracy with an average precision of 0.891 ± 0.015, sensitivity of 0.887 ± 0.011, and F1-score of 0.887 ± 0.013, while maintaining a low false positive rate of $FP_{\mathrm{avg}}$ = 0.83 ± 0.016.

**Table 3:** Lesion-level performance across five folds.

Folds	Precision	Sensitivity (Recall)	F1-score	*FP_avg_*
Fold 1	0.901	0.893	0.897	0.75
Fold 2	0.889	0.884	0.886	0.85
Fold 3	0.873	0.870	0.871	0.97
Fold 4	0.910	0.897	0.903	0.68
Fold 5	0.882	0.893	0.880	0.90
Average	**0.891** ± **0.015**	**0.887** ± **0.011**	**0.887** ± **0.013**	**0.83** ± **0.016**

The bold values indicate the performance of the proposed model.

Notably, the variation across folds was minimal, indicating stable performance regardless of data split. Fold 4 achieved the best results across all metrics, with the highest precision (0.910) and F1-score (0.903), as well as the lowest $FP_{\mathrm{avg}}$ (0.68). These findings support the robustness of the proposed model architecture and the effectiveness of incorporating CBAM into the skip connections for reducing false positives while maintaining sensitivity. The low standard deviation across metrics further suggests that the model can reliably detect cerebral microbleeds across different subsets of the data.

### 4.3 Performance analysis on small lesions ( ≤ 3 mm)

To assess the performance of our model on small cerebral microbleeds (CMBs), we conducted a comparative analysis using lesions with a maximum diameter of 3 mm. Table 4 summarizes the quantitative results across different methods. Our model achieved the highest performance across all key metrics, with a precision of 0.854, sensitivity of 0.883, and F1-score of 0.869. It also reported the lowest average number of false positives per subject (FP_avg = 0.91), indicating effective suppression of incorrect detections.

**Table 4:** Quantitative comparison of lesion-level detection performance for cerebral microbleeds (CMBs) ≤ 3 mm in diameter.

Method	Precision	Sensitivity (Recall)	F1-score	*FP_avg_*
Dou et al. (2016)	0.492	0.731	0.587	5.31
Fan et al. (2022)	0.773	0.754	0.763	2.55
Wei et al. (2022)	0.805	0.849	0.826	1.66
Tsuchida et al. (2024)	0.747	0.717	0.732	1.91
Ours	**0.854**	**0.883**	**0.869**	**0.91**

The bold values indicate the performance of the proposed model.

Compared to the method by Wei et al., our model showed an F1-score improvement of 0.043 and a substantial reduction in FP_avg from 1.66 to 0.91, demonstrating its ability to detect subtle and low-contrast lesions with higher precision. These results suggest that the attention-based skip connection integrated into our model effectively enhances the network’s focus on small lesion features.

Overall, the proposed model provides accurate and reliable detection performance even for tiny lesions, supporting its potential for clinical application in scenarios where high sensitivity and precision are essential.

### 4.4 Qualitative evaluation

**Figure 2:**
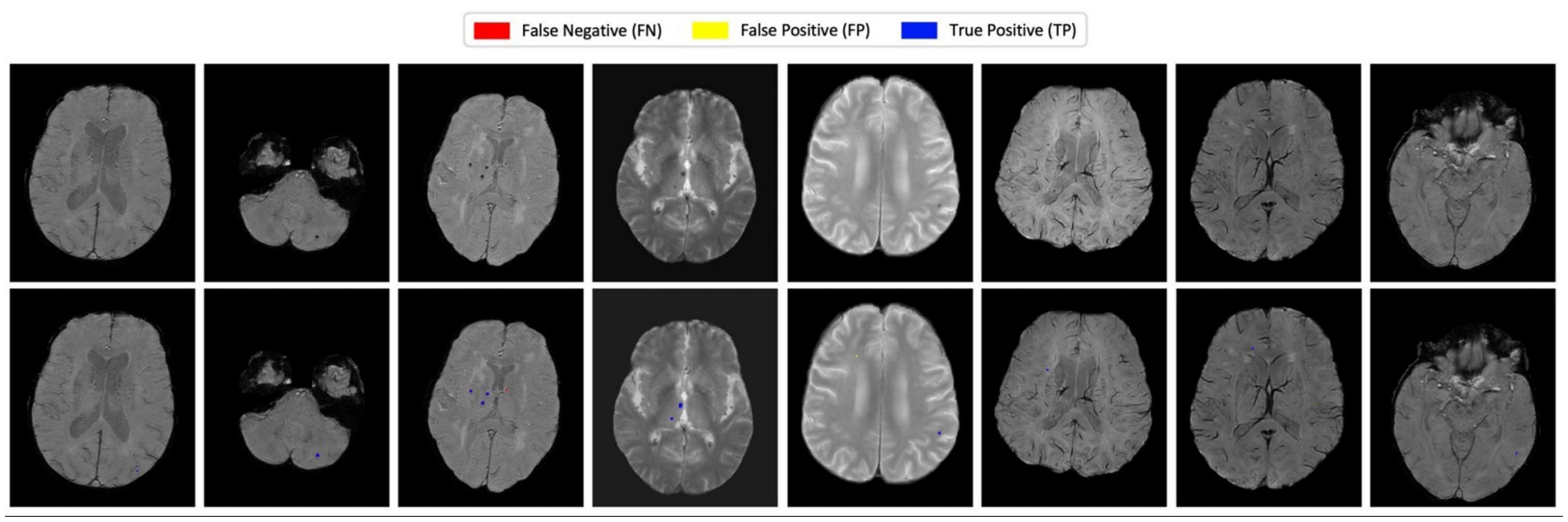
Visual comparison of detection results on representative brain slices. The first row shows original T2*-GRE or SWI images, and the second row displays prediction results. True positives (blue), false positives (yellow), and false negatives (red) are color-coded for visual clarity.

**Figure 3:**
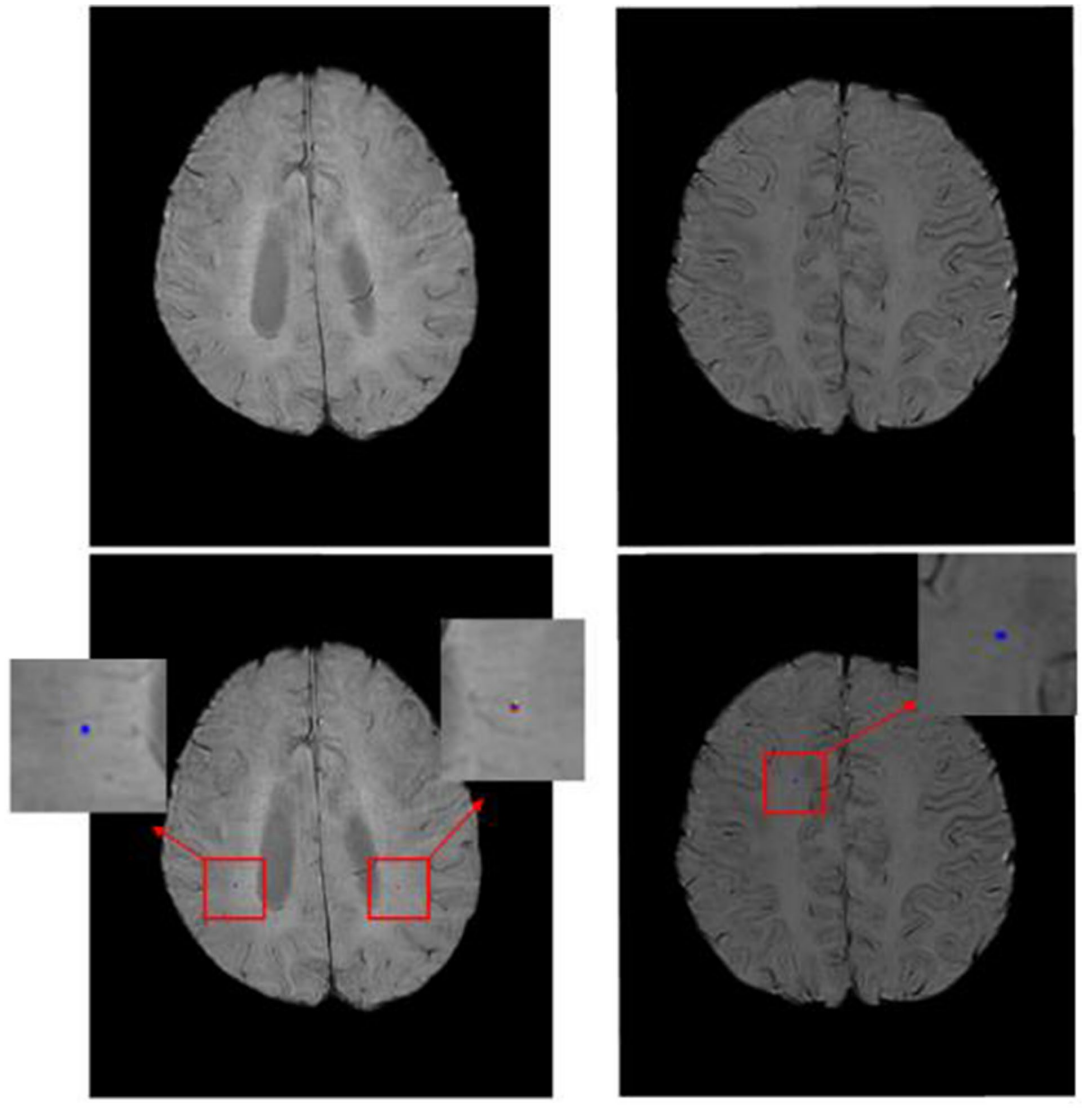
Qualitative visualization of detection results for very small cerebral microbleeds (<3 mm). The examples highlight that the proposed model successfully detects subtle lesions occupying only a few voxels. Although minor discrepancies in lesion size or boundary may occur, the model consistently localizes microbleeds at the correct anatomical sites.

To further evaluate the performance of the proposed model, qualitative visualization results are presented in Figures 2, 3. These figures provide representative examples across a diverse range of imaging appearances and lesion characteristics.

Figure 2 illustrates qualitative detection results for multiple subjects, where each column corresponds to an axial slice from a different case. The top row shows the original T2*-GRE or SWI images without annotations, serving as a reference for image contrast and quality. The bottom row overlays the model’s prediction results, with detections color-coded as follows: true positives (TP, blue), false positives (FP, yellow), and false negatives (FN, red).

As observed in Figure 2, the proposed model successfully detects the majority of cerebral microbleeds with high localization accuracy. Most true positive detections are well aligned with the manually annotated ground truth, indicating precise lesion localization. While minor variations in lesion shape or size may be present, the predicted lesions generally exhibit strong visual correspondence with the ground truth annotations. A small number of false positives are observed, typically arising from low-intensity regions or imaging artifacts that visually resemble microbleeds. False negatives are relatively rare and, when present, are primarily associated with extremely small or faint lesions.

While Figure 2 demonstrates the overall detection behavior of the proposed method, Figure 3 provides additional qualitative examples focusing specifically on very small cerebral microbleeds (<3 mm), which are known to be particularly challenging to detect even for experienced human readers. In these cases, lesions occupy only a few voxels and often exhibit low contrast relative to surrounding tissue.

As shown in Figure 3, the proposed model is able to reliably localize the presence of these very small microbleeds, even when slight discrepancies in lesion extent or boundary definition are observed. Although the predicted segmentation may not perfectly match the precise voxel-level annotations, the model consistently identifies the correct anatomical locations of the lesions. This behavior suggests that the proposed approach prioritizes robust lesion detection over exact boundary delineation in extremely small lesions, which is appropriate for automated CMB screening and burden assessment tasks.

Overall, the qualitative results presented in Figures 2, 3 are consistent with the quantitative findings reported earlier, demonstrating that the attention-enhanced architecture effectively captures subtle CMB patterns while suppressing spurious activations, even in challenging small-lesion scenarios.

### 4.5 Cross-modality generalization between T2*-GRE and SWI

To evaluate the robustness and generalizability of the proposed method across different imaging modalities, we conducted a cross-modality evaluation between T2*-weighted gradient echo (T2*-GRE) and susceptibility-weighted imaging (SWI), as summarized in Table 5. In this experiment, the model was trained exclusively on one modality and tested on the other, thereby assessing its ability to generalize beyond the training domain.

**Table 5:** Cross-modality evaluation results of the proposed method.

Train data	Test data	Precision	Sensitivity (Recall)	F1-score	*FP_avg_*
T2*-GRE	SWI	0.758	0.771	0.765	2.18
SWI	T2*-GRE	0.813	0.827	0.820	1.83

The model was trained on one imaging modality (T2*-GRE or SWI) and evaluated on the other to assess modality-specific robustness and generalization performance.

When trained on T2*-GRE and tested on SWI, the proposed model achieved a precision of 0.758, a sensitivity of 0.771, and an F1-score of 0.765, with an average false positive rate ( $FP_{\mathrm{avg}}$ ) of 2.18 per subject. Conversely, training on SWI and testing on T2*-GRE resulted in improved performance, yielding a precision of 0.813, a sensitivity of 0.827, an F1-score of 0.820, and a reduced $FP_{\mathrm{avg}}$ of 1.83.

The relatively stronger performance observed when training on SWI can be attributed, at least in part, to the higher number of slices typically available in SWI acquisitions, which provides a larger and more diverse set of training samples. This increased data availability may allow the model to learn more robust representations of susceptibility-related features, which subsequently transfer more effectively to T2*-GRE images despite differences in contrast characteristics and artifact profiles.

Importantly, although a moderate performance gap is observed between the two training configurations, the proposed method maintains stable detection performance in both cross-modality settings. This indicates that the model does not rely on modality-specific cues alone, but instead captures modality-invariant characteristics of cerebral microbleeds. Furthermore, when compared with existing CMB detection approaches reported in Table 2, the proposed method demonstrates competitive or superior performance even under cross-modality evaluation, highlighting its strong generalization capability.

Overall, these results suggest that the proposed attention-enhanced architecture exhibits robust cross-modality generalization between T2*-GRE and SWI, supporting its potential applicability in heterogeneous clinical environments where imaging protocols and acquisition settings may vary.

### 4.6 Subject-level prediction of CMB counts aligned with ARIA-H severity framework

To evaluate the clinical relevance and consistency of our model’s performance, we analyzed its ability to estimate the number of cerebral microbleeds (CMBs) per subject using four severity intervals defined by the ARIA-H radiographic classification: 0, 1–4, 5–9, and ≥10 (Hampel et al., 2023). This categorization reflects clinically meaningful thresholds, as outlined in anti-amyloid immunotherapy trials where the CMB burden is used to guide treatment eligibility and safety monitoring. Table 6 presents the subject-level confusion matrix based on this classification scheme.

**Table 6:** Subject-level confusion matrix of predicted versus ground-truth CMB counts categorized by ARIA-H severity intervals.

Prediction GT	0	1–4	5–9	≥10
0	6	2	0	0
1–4	2	46	2	0
5–9	0	2	6	1
≥10	0	0	1	4

The results show that the model’s predictions are predominantly aligned with the ground truth. Among the 50 subjects with 1–4 true CMBs, 46 (92.0%) were correctly categorized. In the 5–9 group, the majority of cases (6 out of 9) were accurately predicted, while minor misclassifications occurred only in adjacent classes (e.g., 1–4 or ≥10), which are clinically tolerable given the gradational nature of severity. Importantly, no subject without CMBs was mistakenly predicted to have a moderate (5–9) or severe (≥10) lesion burden. Similarly, all subjects with ≥10 CMBs were either correctly classified or placed in the adjacent group (5–9), with no cases underestimated into the 1–4 or 0 category.

Such prediction patterns highlight the model’s ability to avoid extreme overestimation or underestimation of CMB burden—an essential quality for reliable clinical deployment. This behavior suggests that the model is not only detecting individual lesions accurately but also maintaining count-level consistency that reflects the true severity of each subject.

We attribute this strength to the CBAM-based skip connection design integrated into our model. While conventional U-Net skip connections may indiscriminately transfer both relevant and irrelevant features—including vessels or background structures—our model leverages spatial and channel attention to selectively enhance lesion-specific representations. This mechanism appears to suppress false positives and improve true lesion detection across a wide spectrum of lesion counts.

Overall, the results demonstrate that our model maintains a clinically aligned, component-level understanding of lesion burden. This capability is particularly critical in the context of ARIA-H–related safety evaluations, where accurate and conservative quantification of microhemorrhages directly impacts trial eligibility and patient risk stratification.

### 4.7 Subject-level correlation analysis for CMB burden estimation

To further assess the reliability of the proposed method at the subject level, we performed a correlation analysis between the predicted and ground-truth cerebral microbleed (CMB) counts on the test set (*n* = 72). For each subject, the total number of detected CMBs was compared with the manually annotated CMB count.

**Figure 4:**
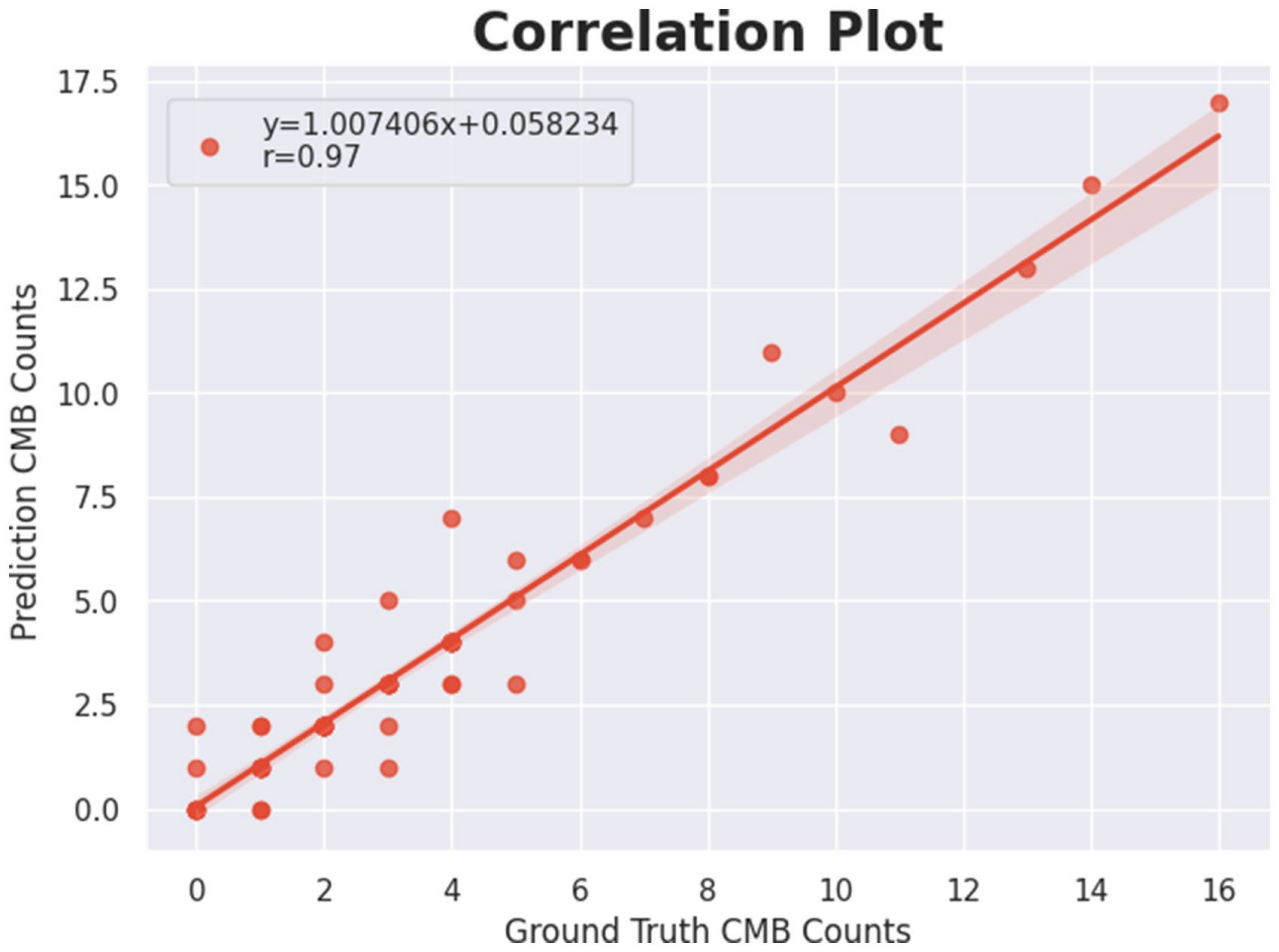
Subject-level correlation between predicted and ground-truth cerebral microbleed counts on the test set (*n* = 72). Each point represents one subject. The regression line and confidence band illustrate the relationship between predicted and true CMB burdens. The Spearman correlation coefficient ( ρ = 0.93) with its 95% confidence interval ([0.890.96]) demonstrates strong and statistically reliable agreement between predicted and ground-truth CMB counts.

As shown in Figure 4, the predicted CMB counts exhibit a strong positive correlation with the ground-truth counts. The Spearman correlation coefficient was ρ = 0.93, indicating a high degree of monotonic agreement between predicted and true CMB burdens. To quantify the statistical reliability of this correlation, a 95% confidence interval (CI) was computed using Fishter z-transformation, yielding a CI of [0.89–0.96] (*p* < 0.001).

In addition to the correlation coefficient, the regression line demonstrates a slope close to unity and a near-zero intercept, suggesting that the proposed model does not systematically overestimate or underestimate the CMB burden across subjects. While minor dispersion is observed in low-burden cases, the overall trend remains consistent across the entire burden range.

These results indicate that the proposed method provides stable and reliable subject-level CMB burden estimation, supporting the robustness of the reported performance beyond single point estimates and reinforcing its potential applicability for clinical CMB severity assessment.

### 4.8 Effectiveness of CBAM: quantitative and visual analysis

To assess the impact of the Convolutional Block Attention Module (CBAM) in our RLK-UNet framework, we conducted a series of ablation experiments focused on both quantitative performance and qualitative attention visualization (Selvaraju et al., 2017).

#### 4.8.1 Quantitative evaluation

Table 7 compares the performance of the model with and without CBAM applied to all skip connections. The inclusion of CBAM yielded a notable improvement across all metrics. Precision increased from 0.783 to 0.891, recall from 0.855 to 0.887, and F1-score from 0.812 to 0.887. Furthermore, the average number of false positives per subject ( $FP_{\mathrm{avg}}$ ) dropped substantially from 1.80 to 0.83, demonstrating the role of CBAM in suppressing irrelevant activations.

**Table 7:** Ablation study comparing the performance with and without CBAM.

Configuration	Precision	Sensitivity (Recall)	F1-score	*FP_avg_*
Without CBAM	0.783	0.855	0.812	1.80
With CBAM	0.891	0.887	0.887	0.83

To further investigate the effect of the number of CBAM modules, we evaluated configurations with CBAM applied to one, two, or all three skip connections. The results are shown in Table 8. As the number of CBAM modules increased, performance consistently improved. Specifically, F1-score improved from 0.850 (CBAM-Skip1) to 0.860 (CBAM-Skip2), and 0.887 (CBAM-AllSkip), while $FP_{\mathrm{avg}}$ decreased from 1.23 to 1.11 and finally 0.83. These findings suggest that the use of CBAM in multiple skip pathways contributes cumulatively to enhanced feature refinement and noise suppression.

**Table 8:** Ablation study of CBAM placement in skip connections.

Configuration	Precision	Sensitivity (Recall)	F1-score	*FP_avg_*
Without CBAM	0.783	0.855	0.812	1.80
CBAM-Skip1	0.841	0.857	0.850	1.23
CBAM-Skip2	0.856	0.864	0.860	1.11
CBAM-AllSkip	0.891	0.887	0.887	0.83

The results show how selectively applying CBAM to 1, 2, or all skip connections influences lesion-level detection performance.

#### 4.8.2 Visual analysis

**Figure 5:**
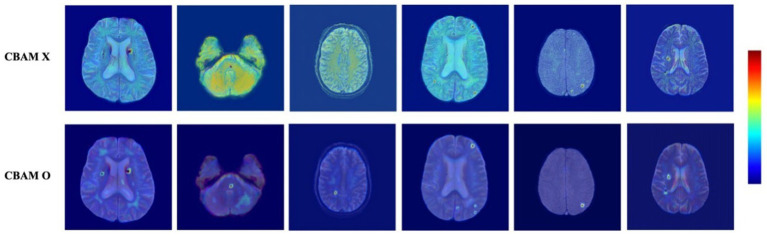
Grad-CAM visualizations comparing models without (top row) and with (bottom row) CBAM. Each pair shows the same axial brain slice. CBAM enhances attention localization near true microbleeds and suppresses irrelevant activations in the background. The color bar indicates activation intensity, ranging from low (blue) to high (red).

**Figure 6:**
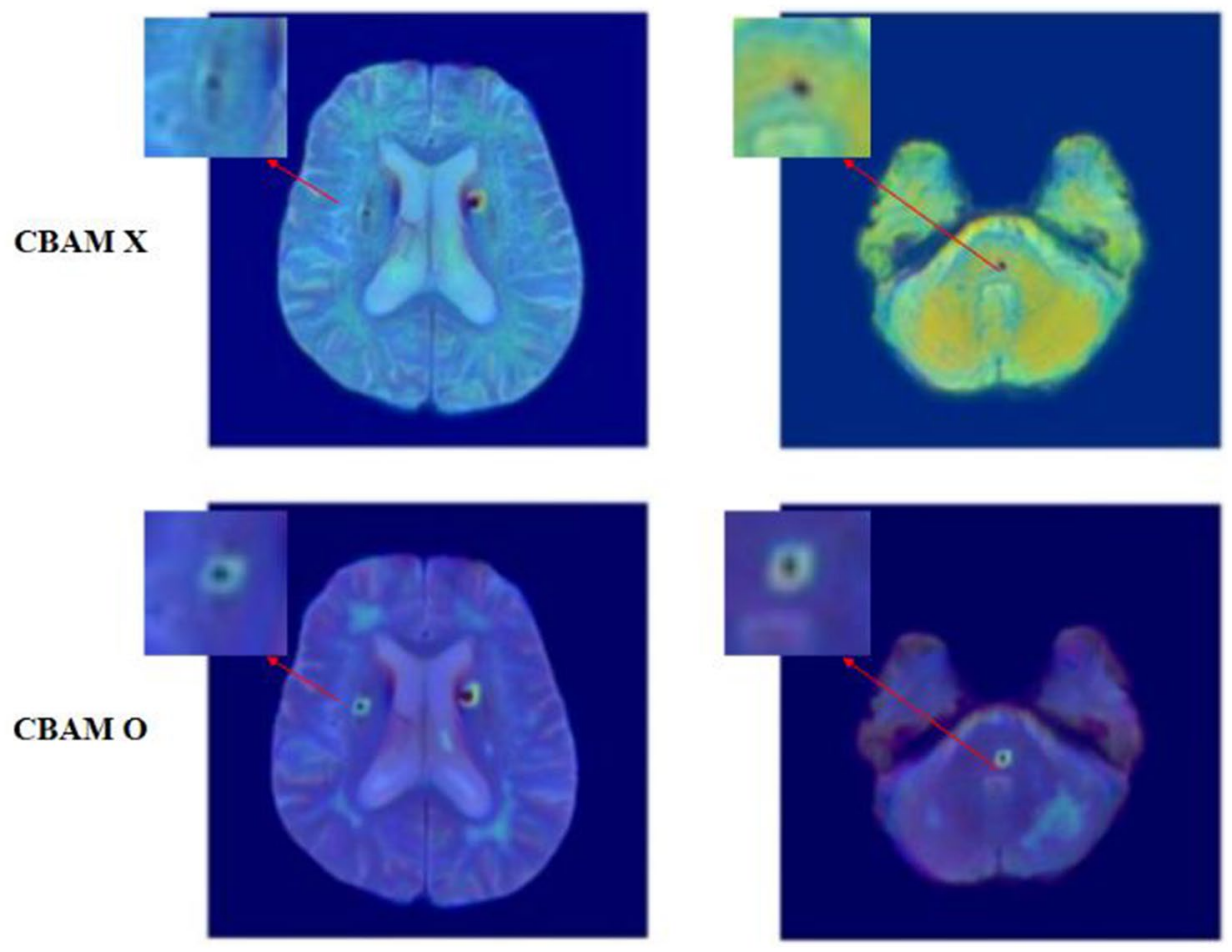
Enlarged Grad-CAM visualizations of small cerebral microbleeds, illustrating differences in attention localization between models without CBAM and with CBAM applied to skip connections.

To explore how CBAM affects spatial attention, we visualized Grad-CAM maps extracted from the final decoder block, comparing the model without CBAM and the model with CBAM applied to all skip connections, as shown in Figures 5, 6.

Figure 5 presents representative Grad-CAM visualizations across multiple subjects. In the absence of CBAM, the attention maps exhibit relatively diffuse activation patterns that frequently extend into non-lesion or background regions, which is consistent with the higher number of false positives observed quantitatively. In contrast, when CBAM is incorporated into the skip connections, the activation maps become more spatially concentrated and selectively focused on lesion-relevant regions, with reduced off-target responses.

To further improve the interpretability of these attention patterns, Figure 6 provides enlarged views of selected cases, highlighting small cerebral microbleeds with additional zoomed-in regions. These focused visualizations allow clearer inspection of the spatial relationship between the Grad-CAM responses and the underlying lesion locations. Even for small and subtle lesions, the CBAM-equipped model consistently demonstrates localized attention centered on the lesion, whereas the model without CBAM shows broader and less specific activation.

Importantly, while slight discrepancies in the exact spatial extent of the highlighted regions may remain, the CBAM-based model reliably identifies the correct anatomical locations of the lesions. This behavior indicates that CBAM enhances the model’s ability to prioritize diagnostically meaningful features rather than diffuse background cues.

Taken together, the quantitative performance improvements and the qualitative Grad-CAM visualizations in Figures 5, 6 confirm that CBAM plays a critical role in improving detection precision and robustness by enhancing spatial and channel-wise feature representation during skip connection fusion.

## 5 Conclusion

In this study, we presented an enhanced deep learning framework for cerebral microbleed (CMB) detection that directly addresses one of the most persistent challenges in this field: the trade-off between sensitivity and precision caused by the visual similarity of CMBs to veins, calcifications, and other susceptibility artifacts. Whereas conventional single-stage segmentation models often propagate these confounding signals through skip connections—achieving higher recall at the expense of precision or vice versa—our approach redefines the skip pathway as a selective, context-filtered channel. By combining large-kernel residual local convolutions, which capture broad contextual cues necessary to distinguish spherical CMBs from elongated vascular structures, with Convolutional Block Attention Modules (CBAM) embedded in every skip connection, the proposed RLK-UNet suppresses noise-related activations and selectively amplifies lesion-relevant features. This design represents not a simple combination of modules but a principled solution to the root cause of false-positive propagation in U-Net–based models.

Comprehensive quantitative evaluation demonstrated that this architecture achieved state-of-the-art performance across all major metrics, with a precision of 0.891, recall of 0.887, F1-score of 0.887, and a markedly reduced average false positive rate ( $FP_{\mathrm{avg}}$ ) of 0.83 per subject. These results significantly outperform prior approaches such as MMOC-Net and SHIVA-CMB, underscoring the effectiveness of context-filtered skip connections for small lesion detection. Ablation experiments confirmed the central role of CBAM in reducing false positives without compromising recall, while Grad-CAM visualizations provided interpretable evidence of how the model suppresses irrelevant structures and concentrates attention on true lesion areas.

Importantly, our model demonstrated clinical relevance by producing subject-level CMB burden estimates that aligned closely with ARIA-H severity categories, a feature directly applicable to the monitoring of patients receiving anti-amyloid therapies. This translational value highlights the potential of our framework not only for research purposes but also as a practical tool for risk stratification, treatment safety evaluation, and long-term disease monitoring in real-world neuroimaging workflows.

Taken together, our findings indicate that RLK-UNet with CBAM offers a robust and clinically meaningful solution for automated CMB detection, balancing precision and sensitivity within a single-stage architecture while maintaining interpretability. In future work, we aim to validate the model on larger, multi-center datasets with diverse acquisition protocols, extend the framework to other small cerebrovascular lesions such as lacunes and enlarged perivascular spaces, and integrate self-supervised learning strategies and uncertainty estimation to further enhance generalizability and clinical trust.

## Data Availability

The data analyzed in this study is subject to the following licenses/restrictions: some of the datasets are private. Requests to access these datasets should be directed to ljm@hanyang.ac.kr.
